# A111 EVALUATION OF REFERRAL COMPLETENESS FOR LARGE NON-PEDUNCULATED POLYPS IN LIGHT OF RECENT INTERNATIONAL CONSENSUS: A SINGLE-CENTER STUDY

**DOI:** 10.1093/jcag/gwae059.111

**Published:** 2025-02-10

**Authors:** B Alabdulkarim, K Khalaf, G May, J Mosko, C Teshima

**Affiliations:** Gastroentertology, University of Toronto, Toronto, ON, Canada; Gastroentertology, University of Toronto, Toronto, ON, Canada; Gastroentertology, University of Toronto, Toronto, ON, Canada; Gastroentertology, University of Toronto, Toronto, ON, Canada; Gastroentertology, University of Toronto, Toronto, ON, Canada

## Abstract

**Background:**

Endoscopic mucosal resection (EMR) is standard-of-care for treating large non-pedunculated colorectal polyps yet often requires referral to expert centers. Hence, inclusion of important information to the therapeutic endoscopist is essential for pre-procedure planning. A recent international consensus statement, comprised of 19 components, aims to improve triage and planning of endoscopic resection for large non-pedunculated colorectal polyps. We sought to determine the current status of inclusion of these reporting elements in referrals to our tertiary center.

**Aims:**

1- Report the rate of complete referrals in light of the international expert consensus statement

2- Investigate the degree of correlation of polyp adjudication between referring endoscopist and local advance therapeutic endoscopiest assessment

3- Explore factors predicting comprehensive reporting

**Methods:**

Single-center review of prospectively collected colorectal polyp referrals for large non-pedunculated polyps from March 2021 to March 2023.

**Results:**

411 referrals for large polyps were received; median size 3 cm; 58% located in ascending colon. 89 % of referrals included the initial assessment date, and only 38% incorporated video or photo documentation, of which 44% were deemed sufficient to demonstrate polyp features. Anatomical location was reported in 96% of referrals, while polyp size was mentioned in only half of the referrals (50%). Polyp morphology was described in 91% as either sessile (n=360) or pedunculated (n=35). Paris classification was reported in 53% of referrals, and LST classification in 90%. 12% of referrals reported four elements of less, while 18% of referrals reported all elements. Correlations with our own endoscopic assessments were diverse, ranging from a robust correlation for anatomical location (r=0.82, 95%CI 0.78-0.86, p < 0.001) to a more modest correlation for Paris classification (r=0.44, 95%CI 0.35-0.52 p < 0.001).

**Conclusions:**

Our study reveals deficiencies in current referral practices and emphasizes the consensus statement’s role in improving the process. Consequences of low-quality referrals may include compromised patient care, leading to delayed diagnoses, inappropriate treatments, and potentially compromised patient outcomes. Thus, we have identified a likely knowledge gap in polyp characterization among referring physicians that will be a focus of a subsequent QI initiative.

**Funding Agencies:**

**None**

